# European women’s perceptions of the implementation and organisation of risk-based breast cancer screening and prevention: a qualitative study

**DOI:** 10.1186/s12885-020-06745-0

**Published:** 2020-03-24

**Authors:** Linda Rainey, Daniëlle van der Waal, Anna Jervaeus, Louise S. Donnelly, D. Gareth Evans, Mattias Hammarström, Per Hall, Yvonne Wengström, Mireille J. M. Broeders

**Affiliations:** 1 Radboud Institute for Health Sciences, Radboud university medical center, PO Box 9101, 6500 HB Nijmegen, The Netherlands; 2 Department of Neurobiology, Care Sciences and Society, Division of Nursing, Karolinska Institutet, Alfred, Nobels allé 23, 23300, 14183 Huddinge, Sweden; 3 Prevent Breast Cancer Research Unit, The Nightingale Centre, Manchester University NHS Foundation Trust, Southmoor Road, Manchester, M23 9LT UK; 4 Genomic Medicine, Division of Evolution and Genomic Sciences, Manchester Academic Health Sciences Centre, Manchester University NHS Foundation Trust, Manchester, M13 9WL UK; 5 The Christie NHS Foundation Trust, Withington, Manchester, M20 4BX UK; 6 Department of Medical Epidemiology and Biostatistics, Karolinska Institutet, Nobels väg 12A, 171 77 Stockholm, Sweden; 7 Department of Oncology, Södersjukhuset, Sjukhusbacken 10, 118 83 Stockholm, Sweden; 8 Theme Cancer, Karolinska University Hospital, Alfred Nobels allé 23, 23300, 14183 Huddinge, Sweden; 9 Dutch Expert Centre for Screening, PO Box 6873, 6503 GJ Nijmegen, The Netherlands

**Keywords:** Breast cancer, Screening, Primary prevention, Risk stratification, Implementation

## Abstract

**Background:**

Increased knowledge of breast cancer risk factors has meant that we are currently exploring risk-based screening, i.e. determining screening strategies based on women’s varying levels of risk. This also enables risk management through primary prevention strategies, e.g. a lifestyle programme or risk-reducing medication. However, future implementation of risk-based screening and prevention will warrant significant changes in current practice and policy. The present study explores women’s perceptions of the implementation and organisation of risk-based breast cancer screening and prevention to optimise acceptability and uptake.

**Methods:**

A total of 143 women eligible for breast cancer screening in the Netherlands, the United Kingdom, and Sweden participated in focus group discussions. The focus group discussions were transcribed verbatim and the qualitative data was analysed using thematic analysis.

**Results:**

Women from all three countries generally agreed on the overall proceedings, e.g. a risk assessment after which the risk estimate is communicated via letter (for below average and average risk) or consultation (for moderate and high risk). However, discrepancies in information needs, preferred risk communication format and risk counselling professional were identified between countries. Additionally, a need to educate healthcare professionals on all aspects of the risk-based screening and prevention programme was established.

**Conclusion:**

Women’s insights identified the need for country-specific standardised protocols regarding the assessment and communication of risk, and the provision of heterogeneous screening and prevention recommendations, monitoring the principle of solidarity in healthcare policy.

## Background

After mammographic screening was shown to effectively reduce breast cancer mortality, most European countries initiated one-size-fits-all population-based screening programmes based on women’s attained age [1]. Over the years, more knowledge of breast cancer risk factors has led to the exploration of risk-based screening, i.e. basing screening policy on a woman’s breast cancer risk. Modifying screening frequency, age range, and modality can reduce both the harms and costs of screening, whilst maintaining the benefits [2–5]. It also enables risk management through primary prevention strategies aimed at known risk factors, such as body weight, alcohol intake (both lifestyle programmes), and breast density (preventative medication).

**Fig. 1:**
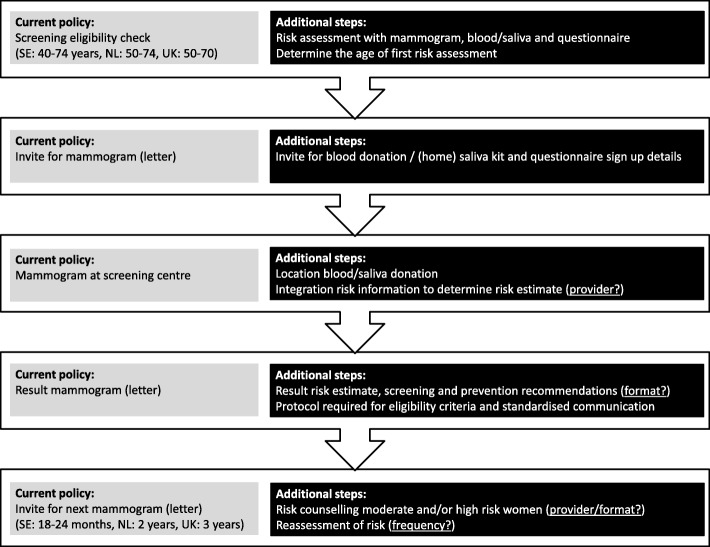
Integration of stages associated with risk-based screening and prevention in a current screening programme

Potential implementation of risk-based breast cancer screening and prevention will require extensive changes in current practice [6]. The programme contains numerous novel stages that will need to be integrated in the current screening infrastructure as illustrated by Fig. 1. For example, the age at which breast cancer risk is assessed should be determined, allowing sufficient time for primary prevention [7, 8]. Furthermore, we need to establish who will assess and relay breast cancer risk and in which format [7]. Certain risk factors (e.g. lifestyle, family history, hormone replacement therapy use) may need to be periodically reassessed and counselling may need to be made available for women with an above average risk of developing breast cancer [7, 8]. Optimal organisation and integration of these additional proceedings will depend on a country’s existing healthcare system and its funding [7].

It has been established that breast cancer risk assessment is feasible in a screening setting and that women are generally interested in knowing their risk [9, 10]. However, women’s preferences regarding the different anticipated procedural pathways of risk-based breast cancer screening and prevention, as described in Fig. 1, have not yet been explored. By keeping women informed and involved in the organisational decision-making process, we can tailor the programme to their needs and facilitate future implementation. Therefore, the present study explores European women’s perceptions of the future implementation and organisation of risk-based breast cancer screening and subsequent primary prevention strategies.

## Methods

### Design

Focus group discussions (FGDs) using a semi-structured interview guide exploring women’s perceptions regarding the organisation of risk-based breast cancer screening and prevention in the Netherlands (NL), the United Kingdom (UK), and Sweden (SE).

### Participants

**Fig. 2:**
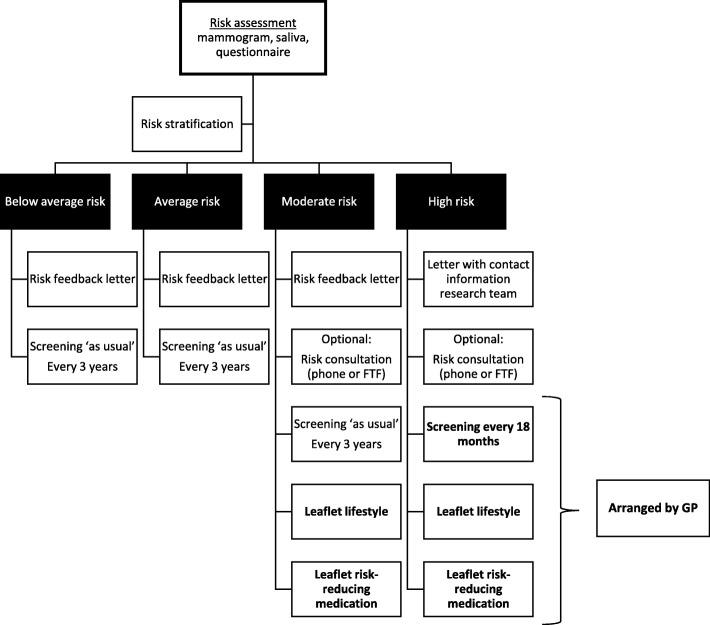
Overview of the PROCAS study procedure

Women were selected from the participant databases of three large prospective cohort studies collecting breast cancer risk information in NL, UK, and SE, i.e. the PRISMA (Personalised Risk-Based Mammography Screening), PROCAS (Predicting the Risk of Cancer at Screening) [11], and KARMA (Karolinska Mammography Project for Risk Prediction of Breast Cancer) study [12], respectively. Participating women from NL (PRISMA) and SE (KARMA) were unaware of their personal breast cancer risk. British participants (PROCAS) had previously been provided with their breast cancer risk and personalised screening and prevention advice between February and December 2016 [9] according to the strategy described in Fig. 2. The recruitment of British participants who had previously received breast cancer risk feedback enabled the unique opportunity to compare hypothetical perceptions about the organisation of risk-based breast cancer screening and prevention, e.g., intent, to actual experiences.

Participants who consented to being approached for follow-up studies were randomly sampled and sent a comprehensive information leaflet. British participants were randomly sampled within their risk category, aiming to invite an equal number of women per category. Women with a previous breast cancer diagnosis were excluded. The Dutch FGDs took place from September – November 2016, the British in February 2017, and the Swedish in April 2017.

### Procedure

The FGDs followed the same semi-structured interview guide in all three countries, which was based on previous research [13, 14] and is available in Supplement 1. Different hypothetical organisational scenarios were discussed with Dutch and Swedish women. Since British women had already received risk feedback and subsequent screening and prevention advice, we did not use hypothetical scenarios, instead evaluating their experiences regarding the organisation of risk-based screening and prevention as regulated by the PROCAS study (Fig. 2). FGDs lasted 60 to 90 min and were performed in the native language of the participants under supervision of one or two moderators with extensive experience in qualitative interviewing (LR, AJ, YW). FGDs were recorded and transcribed verbatim. The Swedish transcripts were translated into English to facilitate independent data analysis. Participants also completed a short questionnaire on demographics and risk perception. FGDs were organised until data saturation was achieved, i.e. no new themes were identified.

### Data analysis and synthesis

The data were thematically analysed per country, independently by pairs of two researchers (LR & DvdW, LR & MB, YW & AJ) using an inductive approach. Six stages were adhered to during data analysis, i.e. familiarisation with the data, coding, developing themes, reviewing themes, defining and naming themes, and final analysis [15]. Consensus was reached through discussion when discrepancies arose.

## Results

### Participant characteristics

We invited 1650 women of whom 143 participated (8.7% response rate) in 20 FGDs. Nine FGDs were conducted in NL (total of 54 participants), five in SE (38 participants), and six in UK (51 participants), with group sizes ranging from 5 to 10 participants. Table 1 provides an overview of participant characteristics. Swedish women were considerably older than the other participants, whereas British women reported the lowest number of years of education. Even though British participants were randomly sampled within their risk category, most of them had a high risk of developing breast cancer (70.6%).

**Table 1:** General characteristics of the study population

Characteristic	Netherlands		Sweden		United Kingdom	
Invited total, N	638		512		500	
Invited per risk category, n (%) (UK only)						
Below average					125	(25.0)
Average					125	(25.0)
Moderate					79	(15.8)
High					171	(34.2)
Participants total, N (response rate, %)	55	(8.6)	38	(7.4)	51	(10.2)
Participants per risk category, n (%) (UK only)						
Below average					–	(−)
Average					10	(19.6)
Moderate					5	(9.8)
High					36	(70.6)
Age^a^ (years), median [range]	57.5	[50–72]	67.0	[44–76]	56.0	[50–69]
Education (years), median [range]	17.0	[6–20]	21.0	[9–21]	15.0	[9–31]
Current employment (% yes)	55.6		42.1		62.7	
Religion (% yes)	42.6		39.5		80.4	
HRT use						
Past (%)	9.3		26.3		15.7	
Current (%)	3.7		7.9		3.9	
Previous breast biopsy (% yes)	9.3		7.9		21.6	
First degree family history breast cancer (% yes)	16.7		21.1		47.1	
Perceived breast cancer risk (%)^b^						
Low	24.1		13.2		3.9	
Below average	18.5		7.9		–	
Average	51.9		55.3		17.6	
Above average	5.6		15.8		54.9	
High	–		2.6		23.5	
Perceived 10-year risk, median [range]	15.0	[0–60]	30.0	[0–75]	50.0	[0–98]

^a^ The eligible screening age in NL is 50–74, SE 40–74 years, and UK 50–70. ^b^ British participants answered the question on perceived risk despite already having received their actual breast cancer risk estimate

### Risk assessment

Women from all three countries were willing to complete a questionnaire, and provide a blood/saliva sample and access to their mammogram to collect breast cancer risk factor information. Most Dutch women would not like an automated risk result after completing a web-based questionnaire. Some Swedish women argued that a web-based questionnaire may limit accessibility. British women emphasised the importance of offering every woman the same risk assessment, after discovering at the FGDs that some women had been asked to provide a saliva sample (for determination of single nucleotide polymorphisms (SNPs)). Women whose risk estimate did not include data on SNPs were doubtful about the accuracy and felt they had not received the complete picture. These procedural differences were dictated by financial restraints, with insufficient research budget available to facilitate the analysis of genetic variation for all PROCAS participants. Since national policy would dictate standardised breast cancer risk assessment, this inconsistency would not occur with potential future implementation.

Most Dutch women favoured a voluntary risk assessment aged 40, enabling women at above average risk to start screening at around 40, with current Dutch screening policy starting at 50. For (below) average risk women they advised an additional mammogram at age 45 to bridge the ten-year interval before screening continuation at 50 to decrease potential anxiety. Most Swedish women concurred that a risk assessment should take place when women turn 40, because they argued that changing your lifestyle becomes easier from that age onwards. British and Dutch women suggested integrating the risk assessment into the cervical screening programme.

### Risk communication

#### Perspective of Dutch and Swedish women based on hypothetical scenarios

Dutch and Swedish women generally agreed that below average and average risk results can be relayed in a letter. Above average risk feedback should be done through a consultation, either via telephone or face-to-face, tailoring the modality to personal preferences indicated at time of consent. Swedish women also suggested group meetings to provide additional information about risk and screening/preventative options. They also recommended the use of modern technology such as video chat. Women from both countries mentioned the GP or a specialised nurse for relaying risk feedback. Dutch women also suggested radiographers employed by the screening centre. Women from both countries also emphasised that they wanted risk feedback from a medical professional with expert knowledge in the field.

Swedish women indicated that they would like to have their risk expressed in a proportion. Additionally, Dutch and Swedish women would like to see their risk represented both in a percentage and visually. Dutch women indicated a preference for recording the risk appointment and both Swedish and Dutch women would like to receive the information in writing to take home. Swedish women stressed that professional support would be required to cope with an above average to high risk result. Women from both countries would like a website or mobile phone application with reliable information about breast cancer risk, screening and prevention. Dutch women also emphasised that professionals need to be adequately informed to prevent the provision of conflicting information about risk and/or preventative options.

#### Evaluating the risk communication procedure with British women

All women, regardless of risk, were satisfied with the format in which their risk was presented to them, i.e. in relative and absolute risks, stating both the chances of getting and not getting breast cancer. Women who were classified as below average to average risk were satisfied receiving their risk in an information letter. Women at moderate to high risk indicated that they appreciated the option of a telephone or face-to-face consultation, although they felt it was sometimes hard to take in all the information due to its emotionally charged nature. They would therefore have appreciated a written report of the consultation.

Some women who were classified as moderate to high risk indicated that the original letter inviting them to get their risk assessed should have expressed more urgency. These women felt that they did not anticipate the consequences of the risk assessment and thought too lightly of it. Another concern for women at moderate to high risk was the knowledge of their GPs. The majority of British FGD participants indicated that their GP was insufficiently informed about tamoxifen/raloxifene and their usage as preventative medication. Consequently, a majority of GPs refused to prescribe the risk-reducing medication, referring women back to the research team. GPs were more willing to continue a prescription, once it had been issued by the local family history consultants under a shared care agreement, which has now become standard practice. Due to this set back, most British women were not in favour of receiving risk feedback and screening and prevention advice from GPs in the future. Instead they recommended the development of special women’s clinics operated by specialised nurses, radiographers, radiologists, and gynaecologists, integrating breast and cervical cancer screening. Additionally, British women signalled a need for pathways and protocols to standardise interaction between primary and secondary care providers to avoid individual variation.

### Accessibility of risk-based screening and prevention

British and Dutch women expressed concern that risk-based screening and prevention may not be equally accessible to all women. The costs of additional mammography in Britain were only covered by the country's National Health Service (NHS) for high-risk women who were under 60 years old. The costs of preventative medication were also not covered. Some Dutch and British women also mentioned potential costs associated with diet and lifestyle changes. They feared that the principle of solidarity in healthcare finance and delivery will be hindered. Therefore, the British and Dutch women called for policy changes to ensure equal access.

### Information needs

An overview of women’s information needs is provided by Table 2. Risk-reducing medication elicited most questions from women in all three countries. However, all stages of the hypothetical risk-based screening and prevention programme elicited a considerable number of questions from Dutch women. Swedish women appeared to have fewer information needs. British women who received a basic level of information at all stages of the programme had few unanswered questions.

**Table 2:** Examples of women’s information needs regarding risk assessment, screening and prevention, stratified by country

Netherlands	Sweden	United Kingdom
***Risk assessment***		
Which factors contribute to my risk?	What factors make up your risk?	I would like to know how risk factors are measured.
How do you calculate risk?	What do they do to assess your risk?	Why weren’t we tested for BRCA?
How reliable is this risk measurement?	What is the risk scale based on?	Which factors specifically contributed to my risk?
Will the risk model be reassessed if a lot of (below) average women still develop breast cancer?	How often can/should you have your risk assessed?	My letter said something about genetic risk, but I didn’t understand it.
What does my risk mean?		How can I lower my risk?
What role do hormones play in breast cancer risk?		
From whom will I receive support if necessary?		
What are the consequences of my risk?		
What are the risk cut-off scores?		
***Screening***		
How can you monitor yourself between mammograms?	How quickly do breast cancers grow?	How reliable are my mammograms if cancers are difficult to detect due to my dense breasts?
At what point should you start worrying (changes to breasts, etc.)?	When does my risk increase sufficiently that I move from average to above average and get more screening?	
What are the risks if you don’t receive biennial screening?		
Provide contact details of professional to contact if you desire screening before allocated interval.		
Is the decreased screening frequency based on scientific evidence?		
Are there other screening modalities for high risk, such as an ultrasound or MRI?		
What are the risks of higher radiation exposure?		
***Lifestyle***		
How much effort is required to decrease you breast cancer risk?	Will my risk be reassessed after I’ve changed my lifestyle?	Missed opportunity that not all women were informed of the link between lifestyle and breast cancer risk.
How can you measure the effects of lifestyle changes on breast cancer risk?	How quickly does your breast cancer risk change after you’ve made lifestyle changes?	
Where can you go for lifestyle advice?		
I need scientific evidence on the link between lifestyle and breast cancer.		
How much do I have to pay if I want to participate in a lifestyle programme?		
By how much can lifestyle decrease your risk?		
I need a unified message on diet.		
Is diet or exercise more effective?		
How do I lower my risk if I already have a healthy lifestyle?		
***Risk-reducing medication***		
How does tamoxifen reduce your risk?	Does tamoxifen have any side effects?	Will there be a follow-up procedure?
Why do I only need to take it for five years?	How does tamoxifen work?	How great is the risk reduction of tamoxifen?
Can I take tamoxifen in combination with other medication?	Are the side effects permanent or do they disappear when you stop taking the medication?	Do the effects of tamoxifen outweigh the risks caused by an unhealthy lifestyle?
Will tamoxifen still work as a treatment for breast cancer when I’ve already used it preventatively?	How do you measure the risk reduction after you’ve started on the tamoxifen?	Will taking tamoxifen for less than five years still be helpful, or do more harm than good?
What is the magnitude of the risk reduction that you can accomplish with tamoxifen?	How many women have taken tamoxifen preventatively?	
What are the short and long term side effects?	Is it safe to stop taking tamoxifen after five years?	
What are alternatives to tamoxifen?	What are alternatives to tamoxifen?	
Is tamoxifen well tested?	How well tested is tamoxifen?	
How much do I have to pay for tamoxifen?	How does tamoxifen affect the menopause?	
Will the risk-reducing effects of tamoxifen last forever?	Is your risk gone after you’ve taken tamoxifen for five years?	
Can the effectiveness of tamoxifen in lowering my risk be measured?		

## Discussion

This study presents the first exploration of Dutch, Swedish, and British women’s perceptions of the potential future implementation and organisation of risk-based breast cancer screening and prevention. There was general agreement between women from the three countries on the overall proceedings, e.g. a risk assessment (consisting of information from a mammogram, questionnaire, blood/saliva) potentially during a cervical screening appointment around the age of 40, after which the risk is communicated via letter or consultation depending on level of risk. However, by comparing hypothetical and actual risk scenarios and focusing on possible culture-specific perceptions, we were able to identify pertinent topics that will need to be addressed before future implementation. It transpires that perceived preferences regarding the organisation of risk-based breast cancer screening and prevention based on hypothetical scenarios (Dutch and Swedish women) do not necessarily correspond to needs and preferences in practice (British women). Dutch and Swedish women’s expressed intent based on their theoretical risk is not always in line with the behaviour of the British women who had received their actual breast cancer risk estimate.

Women from all three countries concurred that a first risk assessment should take place around the age of 40. Although the Swedish screening programme starts screening women at this age, this would mean a policy change for the British and Dutch breast cancer screening programmes that currently start at age 50. Moreover, before women can be categorised into meaningful risk groups, advancements in breast cancer risk prediction are required. Although existing breast cancer risk prediction models, e.g. Tyrer-Cuzick and BOADICEA, perform well on a population level, they lack discriminative accuracy when applied to individual women [16]. By adding other known breast cancer risk factors to these models, e.g. breast density and SNPs, their performance has been shown to moderately improve [17]. Additionally, decisions need to be made about which time frame optimally suits the purpose of the programme, i.e. shorter or longer term risk. British PROCAS participants were relayed their 10-year breast cancer risk, however, a new model has been developed which aims to predict a woman’s short-term risk of breast cancer [18]. This new model integrates a new set of breast cancer risk factors, such as computer-detected microcalcifications and masses visible on the mammogram, resulting in a 2-year breast cancer risk estimate [18]. Although a short-term risk prediction model may provide more accurate risk estimates, a country’s screening interval policy needs to be considered, making the model potentially less applicable to a programme with a 3 year screening interval, like the UK. Moreover, it implies the need for periodical reassessments of risk which would need to be integrated in screening policy.

Adequate understanding of this novel screening and prevention programme is crucial for informed decision-making. First, women need to comprehend the advantages and disadvantages of participating, which some British women felt insufficiently aware of, calling for a more balanced overview in the information leaflets. However, a recent study evaluating the psychological impact of risk communication with PROCAS participants showed no major harms with low anxiety and cancer worry scores after risk communication [19]. After participation, women need to understand their personal risk estimate and subsequent screening and prevention recommendations. Both Dutch and Swedish women struggled to express a preference for a preferred breast cancer risk estimate format, displaying a lack of understanding of both verbal and numerical risk qualifiers. Women from both countries did not understand the implications of being ‘average risk’, attributing more severity to this risk result than ‘in line with current screening assumptions’. Moreover, a considerable number of Dutch women who perceived themselves to be at average risk, translated this risk into having a 50% chance of developing breast cancer within the next 10-years, reasoning average means a 50/50 chance. Although British women did not report a lack of understanding of their 10-year risk estimate, a follow-up study showed great variability in understanding [19]. Moreover, British participants evaluated the positive framing of their risk beneficially, however, this method has been known to lead to risk aversion [20]. This could mean that women will refrain from screening and preventative practices to maintain the perceived risk benefit. British women were not provided with a graphic display of risk, however, Dutch and Swedish women professed a preference for this method. Previous studies of risk communication within a breast cancer screening setting have advised to use verbal, numeral, and visual qualifiers when reporting a person’s cancer risk [21]. Therefore, it may be advisable to further study the added value of a graphic display of risk in a British setting.

Swedish and Dutch women emphasised a role for modern technology in the provision of information, suggesting video chat for risk feedback and counselling, and a telephone application and/or website for up-to-date information on breast cancer, screening, and prevention. This is in accordance with current practice aiming to maximise the quality and efficiency of care by utilising technology in healthcare delivery [22, 23]. However, specific attention will need to be paid to underserved women (e.g. low literacy), since they are less likely to use and communicate through health information technologies [24].

A notable difference in the preferred level of information provision was seen between Dutch and British women. Dutch women wanted comprehensive knowledge of, for example, the measurement properties of the risk prediction model, and the effectiveness of risk-reducing medication and lifestyle changes. These information needs were, however, based on a hypothetical concept of risk-based screening and prevention. British women, who received actual risk feedback and corresponding screening and prevention advice, were satisfied with the relatively basic level of information they were provided with. This may reflect the lower education level of the UK participants. However, the Swedish women who, like the Dutch women, explored their information needs in a hypothetical context, adhered more closely to British women’s information needs. The Swedish women were, on average, highly educated, however, in Swedish society it is relatively uncommon to talk about health risks. This unfamiliarity with the topic may have hindered Swedish participants to brainstorm freely and generate information needs. In general, research shows that the general population struggles to understand information about risk [21]. Information provision will need to aid a woman’s ability to make an informed decision, generally defined as: “A decision where a reasonable choice is made by a reasonable individual using relevant information about the advantages and disadvantages of all the possible courses of action, in accord with the individual’s beliefs” [25]. An exhaustive amount of complicated information on risk and the scientific background of screening and preventative options may hinder this process. The current information leaflet that British, Dutch, and Swedish women receive with their screening invitation is already rather extensive with detailed information about breast cancer, the screening procedure, and the potential benefits (early detection) and risks of screening (false-positives, overdiagnosis). Nevertheless, the information needs identified by the present study can be used to develop country-specific communication tools to potentially inform women in the future about the new programme and their breast cancer risk with subsequent screening and prevention recommendations. The development of a decision aid may assist women in making an informed decision about participation.

British women identified a great need to educate professionals, GPs in particular, on all aspects of the risk-based screening and prevention programme to ensure consistent information provision. Although this study showed that the preferred professional to provide risk feedback and counselling differs per country and depends on existing care pathways, it is probable that a gap in knowledge will exist [6]. This has previously been reported by primary care providers who experienced insufficient training when informing women about their breast cancer risk and subsequent screening and prevention recommendations [26–29]. They also indicated a need for more consultation time, more time to monitor progress, and clear guidelines on the prescription of preventative behaviours (e.g. risk-reducing medication, lifestyle changes) [6]. These additional needs may also apply to healthcare professionals providing risk-based breast cancer screening and prevention counselling. Therefore, training modules will need to be offered to professionals to increase their ability and perceived competence. Standardised protocols regarding the assessment and communication of risk, and the provision of additional screening and risk-reducing medication need to be developed. Crucially, sufficient time needs to be allocated to professionals to be able to meet the information needs of women, potentially allowing for audio recording of the consultation.

British, Dutch, and Swedish women’s perceptions of the organisation of risk-based breast cancer screening and prevention generally appear in line with those of healthcare professionals. This is a good starting point, however, for future implementation these perceptions will need to be integrated into countries’ existing healthcare policies, taking into account current regulations and available resources. Additionally, the current screening pathway will need to remain available for women who do not want to know their breast cancer risk. Furthermore, the mass assessment and storage of sensitive (genetic) risk information will require the development of new moratoria to regulate accessibility and usage [30]. Meanwhile, the principle of solidarity in healthcare policy needs to be monitored, watching out for a discrepancy in healthcare recommendations and insurance coverage.

### Strengths and limitations

To our knowledge, this is the first study to qualitatively explore women’s perceptions of the future implementation and organisation of risk-based breast cancer screening and prevention. Moreover, the participation of a substantial number of women from three different European countries enables international comparisons and insights into ways to optimise women’s informed decision-making and uptake. However, although the present study provides relevant insights, it is not always clear whether differences in perceptions are due to cultural aspects or the ‘hypothetical’ versus ‘actual’ nature of the risk scenarios. We therefore have to be careful when specifically attributing differences in women’s perceptions to cultural variety.

Furthermore, we have identified the perceptions of a relatively homogeneous and selective sample of women. Focus group attendees had previously participated in both screening and scientific research on risk-based breast cancer screening and prevention, i.e. the KARMA, PRISMA, or PROCAS study. Additionally, we had limited to no response from women with a low socioeconomic status or British women at (below) average risk. Our focus group discussions served as a first exploration of women’s perceptions of the organisation of risk-based breast cancer screening and prevention; a topic that has not been widely studied. With our qualitative study we gained a rich, contextualised understanding of a topic through intensive study of particular cases [31]. Our in-depth results are especially well suited for revealing higher-level concepts and theories that are not unique to individual participants or settings [31]. Additional quantitative research with larger groups of women is required to confirm our findings.

## Conclusions

Integration of risk-based breast cancer screening and prevention is dependent on a country’s existing healthcare policy and care pathways. Women’s insights revealed that country-specific standardised protocols for the assessment and communication of breast cancer risk, and the provision of heterogeneous screening and prevention recommendations need to be developed. Additional training needs to be made available for participating healthcare professionals. The principle of solidarity in healthcare policy needs to be monitored, taking into account women who do not want to know their breast cancer risk.

## Supplementary information

**Additional file 1.** Semi structured interview guide used for focus group discussions in all three countries.

## Data Availability

The qualitative data generated and analysed during the current study are not publicly available due to extensive nature of the focus group transcripts, but are available from the corresponding author on reasonable request.

## Abbreviations

FGD: Focus group discussion
GP: General practitioner
KARMA: Karolinska Mammography Project for Risk Prediction of Breast Cancer
NL: the Netherlands
PRISMA: Personalised Risk-Based Mammography Screening
PROCAS: Predicting the Risk of Cancer at Screening
UK: United Kingdom
SE: Sweden
SNP: Single nucleotide polymorphism
