# Supplementary material for: European women’s perceptions of the implementation and organisation of risk-based breast cancer screening and prevention: a qualitative study
Source: BMC Cancer. 2020 Mar 24;20:247. doi: 10.1186/s12885-020-06745-0 (PMC7092605; doi:10.1186/s12885-020-06745-0)
Supplement: Supplementary file 1 — Additional file 1. Semi structured interview guide used for focus group discussions in all three countries. [file 12885_2020_6745_MOESM1_ESM.docx]

**Semi-structured interview guide focus group discussions**

*“Women’s perceptions on personalised risk-based breast cancer screening and primary prevention”*

*Before we start we would like to emphasise that all information will be treated as strictly confidential and your identity will not be revealed in any reports. The recordings will be kept securely under lock and key.*

**Suggested topics** *general*

1. Would you like to know your personal risk of developing breast cancer?
2. Would knowing your personal risk influence your participation in the screening programme?
3. How do you think you’d feel about receiving personal risk information?
4. What would you like about receiving personal risk information/ what would be the best part?
5. What would you dislike about receiving personal risk information?
6. Who do you think should determine your personal breast cancer risk?
7. Who do you think should inform you of your personal breast cancer risk?
8. How do you feel you should be informed of your risk (letter, telephone, face-to-face)?
9. When do you think you should be informed of your personal breast cancer risk?
10. How do you feel about actively working on reducing your risk?
    1. How do you feel about taking risk-reducing medication?
    2. How do you feel about altering your lifestyle?
11. How do you think personalised risk-based screening could benefit you?
12. How do you think breast cancer prevention options could benefit you?
13. What do you think the drawbacks of personalised screening could be for you?
14. What do you think the drawbacks of primary prevention could be for you?
15. What information would you need about personalised risk-based screening to make an informed decision about screening/prevention?
16. What information would you need about prevention to make an informed decision?
    1. Risk reducing medication
    2. Lifestyle alterations
17. How would you feel if your neighbour/friend/relative has a different screening strategy to you?
18. How would you feel about providing a blood sample to determine your breast cancer risk?
19. Which new screening strategy would be acceptable to you:
    1. Screen women aged 35-50 at above average risk, and all women who were already eligible
    2. Only screen women aged 35 years and older who are at above average risk
    3. Other options...

*We are now going to tell you about Delia. We would like to ask you to listen carefully and try to put yourself in Delia’s place. Imagine what you would think and feel if you were in her position.*

**Vignette 1**: Delia is a 58 year old woman. She is told that her risk of developing breast cancer is lower than average. Delia is therefore advised to decrease her screening frequency from once every 3 years to once every 5 years?

**Suggested topics** *Personalised risk-based breast cancer screening*

1. How would you feel if you were in Delia’s shoes?
2. What thoughts does the low risk result provoke?
3. What feelings does the low risk result provoke?
4. What thoughts does the advice on screening frequency provoke?
5. What feelings does the advice on screening frequency provoke?
6. What impact would receiving this risk information have on your life?
7. How do you think this screening advice could benefit you?
8. What do you think the drawbacks of this screening advice could be for you?
9. What information on personalised screening would you need to be able to make an informed choice?
10. How would you like the information on your personal breast cancer risk presented to you?
11. What support would you like to be able to make an informed choice about altering your screening strategy?
12. Would you like to involve other people in the decision making process?
13. How would you feel if your neighbour/friend/relative received mammograms more frequently compared with you?

**Suggested topics** *Primary prevention*

1. Do you feel Delia should be advised to change her lifestyle to further lower her breast cancer risk?

*Next, we are going to tell you about Patricia. Again, we would like to ask you to listen carefully and put yourself in Patricia’s place. Imagine what you would think and feel if you were in her position.*

**Vignette 2:** Patricia is a 50 year old woman. She is told that her risk of developing breast cancer is in the high-risk category. Patricia is advised to increase her screening frequency to once a year. In addition, she is offered the risk reducing medication called Tamoxifen. Tamoxifen is a tablet medication which Patricia would have to take daily for 5 years.

**Suggested topics** *Personalised risk-based breast cancer screening*

1. How would you feel if you were in Patricia’s shoes?
2. What thoughts does the high risk result provoke?
3. What feelings does the high risk result provoke?
4. What thoughts does the advice on changing screening age and frequency provoke?
5. What feelings does the advice on changing screening age and frequency provoke?
6. What impact would receiving this risk information have on your life?
7. How do you think this screening advice could benefit you?
8. What do you think the drawbacks of this screening advice could be for you?
9. What information on personalised screening would you need to be able to make an informed choice?
10. How would you like the information on your personal breast cancer risk presented to you?
11. What support would you like to be able to make an informed choice about altering your screening strategy?
12. Would you like to involve other people in the decision making process?

**Suggested topics** *Primary prevention*

1. What thoughts does the advice on Tamoxifen provoke?
2. What feelings does the advice on Tamoxifen provoke?
3. How do you think this prevention advice could benefit you?
4. What do you think the drawbacks of this prevention advice could be for you?
5. What information on Tamoxifen would you need to be able to make an informed choice?
6. How would you like the information on Tamoxifen presented to you?
7. Who do you think should inform you about Tamoxifen?
8. What support would you like to be able to make an informed choice about Tamoxifen?
9. Would you like to involve other people in the decision making process?

*Finally, we would like to introduce you to Mary. Please listen carefully and imagine what you would think and feel if you were in Mary’s position.*

**Vignette 3:** Mary is 50 years old, she is told that she has an average risk for developing breast cancer. She is told that her screening strategy will remain as it is. She will start screening at 50 years and will be invited every three years. She is advised to change her lifestyle to further reduce her breast cancer risk. The following lifestyle choices are recommended:

- Obtain and maintain a healthy weight; Mary is currently somewhat overweight
- Eat a healthy Mediterranean diet
- Participate in moderate physical exercise 3-4 hours a week
- Reduce alcohol intake to 1 glass a day

**Suggested topics** *Personalised risk-based breast cancer screening*

1. How would you feel if you were in Mary’s shoes?
2. What thoughts does the average risk result provoke?
3. What feelings does the average risk result provoke?
4. What thoughts does the advice on screening provoke?
5. What feelings does the advice on screening provoke?
6. What impact would receiving this risk information have on your life?
7. How do you think this screening advice could benefit you?
8. What do you think the drawbacks of this screening advice could be for you?
9. What information on personalised screening would you need to be able to make an informed choice?
10. How would you like the information on your personal breast cancer risk presented to you?
11. What support would you like to be able to make an informed choice?
12. Would you like to involve other people in the decision making process?
13. How would you feel if your neighbour/friend/relative received mammograms less frequently compared with you?

**Suggested topics** *Primary prevention*

1. What thoughts does the advice on lifestyle choices provoke?
2. What feelings does the advice on lifestyle choices provoke?
3. How do you think this prevention advice could benefit you?
4. What do you think the drawbacks of this prevention advice could be for you?
5. What information on lifestyle choices would you need to be able to make an informed choice?
6. How would you like the information on lifestyle choices presented to you?
7. Who do you think should inform you about lifestyle choices?
8. What support would you like to be able to make an informed choice about lifestyle changes?
9. Would you like to involve other people in the decision making process?
